# Sugarcane cutting work, risks, and health effects: a literature review

**DOI:** 10.11606/S1518-8787.2018052000138

**Published:** 2018-08-23

**Authors:** Marceli Rocha Leite, Dirce Maria Trevisan Zanetta, Iara Buriola Trevisan, Emmanuel de Almeida Burdmann, Ubiratan de Paula Santos

**Affiliations:** I Divisao de Pneumologia, Instituto do Coracao, Hospital das Clinicas HCFMUSP, Faculdade de Medicina, Universidade de Sao Paulo, Sao Paulo, SP, BR; II Universidade de São Paulo. Faculdade de Saúde Pública. Departamento de Epidemiologia. São Paulo, SP, Brasil; III Universidade Estadual Paulista “Júlio de Mesquita Filho”. Departamento de Fisioterapia. Campus de Presidente Prudente. São Paulo, SP, Brasil; IV Universidade de São Paulo. Faculdade de Medicina. Hospital das Clinicas. Divisão de Nefrologia. São Paulo, SP, Brasil

**Keywords:** Rural Workers, Health Status, Working Conditions, Occupational Risks, Occupational Health, Review, Trabalhadores Rurais, Nível de Saúde, Condições de Trabalho, Riscos Ocupacionais, Saúde do Trabalhador, Revisão

## Abstract

**OBJECTIVE:**

Describe the main work risks for sugarcane cutters and their effects on workers’ health.

**METHODS:**

Critical review of articles, with bibliographic research carried out in the PubMed, SciELO Medline, and Lilacs databases. The following keywords were used: sugarcane workers, sugarcane cutters, sugarcane harvesting, *cortadores de cana-de-açú* *car* , and *colheita de cana* *-de-açúcar* . The inclusion criteria were articles published between January 1997 and June 2017, which evaluated working conditions and health effects on sugarcane cutters. Those that did not deal with the work impact of cutting burned and unburnt sugarcane in the cutter’s health were excluded. The final group of manuscripts was selected by the lead author of this study and reviewed by a co-author. Disagreements were resolved by consensus using the predefined inclusion and exclusion criteria and, where necessary, the final decision was made by consulting a third co-author.

**RESULTS:**

From the 89 articles found, 52 met the selection criteria and were evaluated. Studies have shown that cutters work under conditions of physical and mental overload, thermal overload, exposure to pollutants, and are subject to accidents. The main effects observed were respiratory, cardiovascular, renal, musculoskeletal, heat stress, dehydration, genotoxic, and those due to accidents.

**CONCLUSIONS:**

Work on the manual cutting of sugarcane, especially of burned sugarcane, exposes workers to various risks, with different health impacts. Risk reduction for exposure to pollution and thermal and physical overload is required as a measure to preserve the health of the worker.

## RESUMO

**OBJETIVO:**

Descrever os principais riscos do trabalho de cortadores de cana-de-açúcar e seus efeitos na saúde dos trabalhadores.

**MÉTODOS:**

Revisão critica de artigos, com pesquisa bibliográfica realizada nas bases de dados PubMed, SciELO Medline, e Lilacs. Foram empregadas as palavras-chave: *sugarcane workers,* *sugarcane cutters, s* *ugarcane harvesting* , cortadores de cana-de-açúcar e colheita de cana-de-açúcar. Os critérios de inclusão foram artigos publicados entre janeiro de 1997 a junho de 2017, que avaliaram as condições de trabalho e os efeitos na saúde em trabalhadores cortadores de cana-de-açúcar. Foram excluídos aqueles que não tratassem do impacto do trabalho no corte da cana-de-açúcar queimada e não queimada na saúde do cortador. O grupo final de manuscritos foi selecionado pelo autor principal deste estudo e revisado por um coautor. As discordâncias foram resolvidas por consenso usando os critérios de inclusão e exclusão predefinidos e, quando necessário, a decisão final foi realizada consultando um terceiro coautor.

**RESULTADOS:**

De 89 artigos encontrados, 52 atenderam aos critérios de seleção e foram avaliados. Os estudos mostraram que os cortadores trabalham em condições de sobrecarga física e mental, sobrecarga térmica, exposição a poluentes e sujeitos a acidentes. Os principais efeitos observados foram respiratórios, cardiovasculares, renais, osteomusculares, estresse por calor, desidratação, genotóxicos e decorrente de acidentes.

**CONCLUSÕES:**

O trabalho no corte manual da cana-de-açúcar, principalmente da cana-de-açúcar queimada, expõem os trabalhadores a diversos riscos, com variados impactos à saúde. A redução de riscos com exposição à poluição e sobrecarga térmica e física se impõe como medida para preservação da saúde do trabalhador.

## INTRODUCTION

Sugarcane is widely cultivated in Latin America, in Asia, and in Brazil, the world’s largest producer ^1^ . Because of the oil crisis, ethanol production in Brazil gained momentum as fuel used in automotive vehicles and reduced the cost of petroleum products imports starting in 1970. There was a large increase in sugarcane production in the country to replace fossil fuels with biofuels, in addition to meeting the demand for sugar ^2^ ^,^ ^3^ . This new scenario boosted the development of new production regions in the state of São Paulo and in the Northeast region, and expanded production to Paraná, Goiás, Mato Grosso, and Mato Grosso do Sul. The growth of sugarcane production in Brazil (768 million tons of sugarcane in the 2016 harvest), also occurs in Asian countries, most notably in India (348 million tons), China (123 million tonnes), and Thailand (87 million tonnes) ^1^ .

The manual harvesting of sugarcane has used the practice of burning straw to facilitate manual cutting, reducing water content and thereby increasing sugar content, as well as eliminating venomous animals ^4^ . However, the burning of sugarcane straw is responsible for the emission of large quantities of pollutants that contribute to adverse effects on the health ^5^ of workers and populations of cities near the burning regions. The practice of burning, although it occurs in several countries, is more widespread in Brazil. The country ranked first in the emissions of biomass burning from sugarcane in 2016 (6.6 million tons), followed by India and China (3.2 and 1.1 million, respectively) ^6^ .

State Law 11,241/2002 was approved in the State of São Paulo, after pressure from environmental movements, researchers, and the population of affected cities. This law gradually banned the burning of sugarcane, seeking to end it by 2031. With the increase of pressures, the Agro-Environmental Protocol of the State of São Paulo was signed in 2007, which anticipated the deadlines for eliminating the practice of burning in 2014 in flat areas and 2017 in rough terrain ^7^ . However, the burning of sugarcane prior to manual cutting is still performed in some regions of Brazil and in several countries, even with proven adverse effects ^7^ .

In the last 20 years, there have been studies that evaluated the working conditions and health effects of rural cane cutters. During manual cutting, workers are exposed to a number of health hazards, such as: physical hazards – weather conditions (high temperatures, solar radiation, rain), noise emitted by vehicles; chemical hazards – gases and particulate matter from burning cane, soil, and pesticide residues; biological hazards – venomous animals; risks of accidents: traumas and fire; ergonomic risks – repetitive postures and movements, physical overload, and mental risks imposed by the work rhythm, constant attention, concentration, and lack of regular pauses ^8^ ^,^ ^9^ . Studies conducted in Central American countries with sugarcane cutters also show high morbidity and mortality, mainly associated with the epidemic of chronic kidney disease^10–12^. Despite several studies, there is a lack of systematization of the findings and evidence, as well as of the suggested measures to preserve the health of the workers.

This review aimed to describe the risks of sugarcane cutters’ work and its effects on workers’ health.

## METHODS

Bibliographic search in databases: PubMed, SciELO, Medline, and Lilacs. The inclusion criteria were: articles published in Portuguese and English, between January 1997 and June 2017, during which time a greater number of studies that could be accessed in full were published. The articles were selected based on the analysis of their titles, followed by their abstract. Those that did not deal with the work impact of cutting burned and unburnt sugarcane in the cutter’s health were excluded. The keywords used to search the databases were: sugarcane workers, sugarcane cutters, sugarcane harvesting, *cortadores de cana-de-açúcar* , and *colheita de cana-de-* *açúcar* . The final set of manuscripts was selected by the lead author and reviewed by a co-author. Disagreements were resolved by consensus using the predefined inclusion and exclusion criteria and, where necessary, the final decision was made by consulting a third co-author.

Of the 89 articles evaluated, 13 were discarded after the title analysis, 22 after reading the abstract or the full text, and two because they were not accessible in their entirety, leaving 52 articles included ( Figure ).

**Figure:**
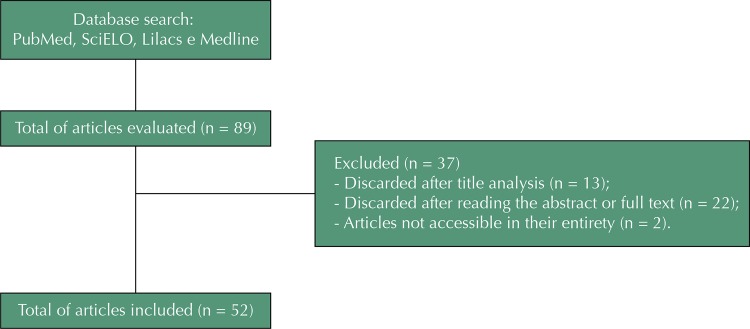
Flowchart of the selection of articles for review.

## RESULTS AND DISCUSSION

The list of 52 evaluated articles, grouped according to the topics discussed, is shown in Box 1 and 2 and are detailed below.

**Box 1:** List of topics covered in the studies evaluated.

Topics covered	Authors	Number of articles
Mucociliary clearance	Ferreira-Ceccato et al. ^20^ ; Goto et al. ^19^	2
Respiratory symptoms	Prado et al. ^18^ ; Ferreira-Ceccato et al. ^20^	2
Pulmonary function	Prado et al. ^18^ ; Goto et al. ^19^	2
Inflammatory markers and oxidative stress	Prado et al. ^18^ ; Barbosa et al. ^14^ ; Santos et al. ^31^	3
Pulmonary disease	Sacchi et al. ^79^	1
Cardiovascular effects	Barbosa et al. ^14^ ; Vilela et al. ^13^	2
Renal effects	Garcia-Trabanino et al. ^45^ ; Laws et al. ^58^ ; Laws et al. ^59^ ; López-Marin et al. ^60^ ; Murray et al. ^12^ ; Peraza et al. ^41^ ; Roncal-Jimenez et al. ^61^ ; Santos et al. ^31^ ; Wesseling et al. ^62^ ; Wesseling et al. ^11^ ; Wesseling et al. ^63^ ; Wijkström et al. ^46^	12
Miscellaneous infections (toxoplasmosis, leptospirosis, and brucellosis)	Adesiyun et al. ^64^	1
Quality of life assessment	Carvalho-Junior et al. ^65^	1
Stress, physical and mental symptoms	Priuli et al. ^66^	1
Frequency of sick leave due to occupational diseases	Ferreira-Ceccato et al. ^67^	1
Ergonomic assessment of the work	Abrahão et al. ^16^	1
Occurrence of fungi in the ocular conjunctiva	Dalfré et al. ^68^	1
Risk of lung and oral cavity cancer	Amre et al. ^69^ ; Coble et al. ^70^	2
Exposure to potentially carcinogenic agents and genotoxic effects	Prado et al. ^18^ ; Bosso et al. ^47^ ; Martinez-Vanezuela et al. ^48^ ; Silveira et al. ^71^	4
Work process on the health of sugarcane cutters	Alessi et al. ^55^ ; Alves ^17^ ; Galiano et al. ^72^ ; Moraes et al. ^73^ ; Ribeiro ^8^ ; Rocha et al. ^57^ ; Rocha et al. ^9^ ; Rosa e Navarro. ^74^ ; Scopinho et al. ^75^ ; Vilela et al. ^13^ ; Bitencourt et al. ^49^	11
Musculoskeletal disorders	de Anchieta Messias et al. ^56^ ; Phajan et al. ^37^	2
Thermal overload	Bodin et al. ^50^ ; Crowe et al. ^38^ ; Crowe et al. ^15^ ; Crowen et al. ^1^ ^0^; Roscani et al. ^51^ ; Vilela et al. ^13^	6
Body composition/hydration and nutritional status	Cortez et al. ^52^ ; Chiarello et al. ^53^ ; Florêncio et al. ^76^ ; Luz et al. ^77^ ; Luz et al. ^54^	5
Dermatological changes	Miranda et al. ^78^	1

**Box 2:** Articles analyzed according to authors, year of publication, objectives, and main results.

Authors/Study	Study	Results
Ferreira-Ceccato et al. ^20^ (2011) Panel study, with repeated measurements.	Nasal mucociliary clearance in 45 sugarcane cutters State de São Paulo, Brazil	Reduction of the transit time of saccharin, on the first day of harvest compared to the pre-harvest period.
Goto et al. ^19^ (2011) Panel study, repeated measurements	Nasal mucociliary clearance of 27 sugarcane cutters State de São Paulo, Brazil	Increased in the transit time of nasal mucus after six months of harvest.
Prado et al. ^18^ (2012) Panel study, repeated measurements	Respiratory symptoms, lung function, markers of oxidative stress, and exposure to aromatic hydrocarbons in 113 cutters of burnt sugarcane State de São Paulo, Brazil	Higher incidence of respiratory symptoms, decreased pulmonary function, oxidative stress, increase of 1-hydroxypyrene (1-OH) during the harvest, compared to the pre-harvest period.
Sacchi et al. ^79^ (2013) Case-control study	Risk factors for pulmonary and extrapulmonary tuberculosis among indigenous peoples in Brazil	Indigenous workers in sugarcane plants are 6.8 times more likely to have tuberculosis.
Barbosa et al. ^14^ (2012) Panel study, repeated measurements	Effects of burned cane harvest on blood markers and cardiovascular system in 28 sugarcane cutters State de São Paulo, Brazil	Increase in blood pressure and blood coagulability during the harvest period compared to the pre-harvest period.
Garcia-Trabanino et al. ^45^ (2015) Cross-sectional study	Thermal stress, dehydration, biomarkers of renal function and their possible associations in 189 sugarcane cutters of El Salvador	The average temperature of the working day was 34 °C-36 °C before noon and 39 °C-42 °C at noon. There was a reduction in the glomerular filtration rate in 14% of the workers, and an increase in serum creatinine, uric acid, and urea.
Laws et al. ^58^ (2015) Longitudinal study	Changes in renal function over six months of sugarcane harvest in 284 Nicaraguan workers	Decreased renal function during the harvest.
Laws et al. ^59^ (2016) Prospective cohort study.	Changes in renal injury markers of 284 Nicaraguan rural workers	Of the seven categories of workers studied, sugarcane cutters presented a higher risk of renal damage.
López-Marin et al. ^60^ (2014) Cross-sectional study	Histopathological characterization of chronic kidney disease (CKD) in patients from agricultural communities, including sugarcane cutters El Salvador	Presence of chronic tubulointerstitial nephropathy with glomerular and secondary vascular injury.
Murray et al. ^12^ (2015) Review study	Evaluation of potential pathogens responsible for Mesoamerican nephropathy in Nicaragua	Infectious pathogens present in urine and rodent feces are thought to expose workers during the cultivation and harvesting of cane and are associated with CKD.
Peraza et al. ^41^ (2012) Cross-sectional study	Prevalence of decreased renal function in men and women (farmers) in five communities in El Salvador	Agricultural work on sugarcane and cotton plantations was associated with decreased renal function.
Roncal-Jimenez et al. ^61^ (2015) Cross-sectional study	Increased uric acid concentrations as a possible cause of kidney damage. Review and pilot study evaluating 10 sugarcane cutters in El Salvador	Serum levels of uric acid increased after a work shift and reached hyperuricemia (≥ 7.0 mg/dL).
Santos et al. ^31^ (2014) Longitudinal panel study with repeated measurements	Effect of work cutting burned sugar cane on the renal function of 28 workers State de São Paulo, Brazil	Decreased glomerular filtration rate at the end of the work shift, in all evaluated patients, and, in 18.5% of them, the increase in serum creatinine was consistent with acute kidney injury (AKI).
Wesseling et al. ^62^ (2015) Review study	Risk assessment of CKD mortality in rural workers Costa Rica	Mortality due to CKD in men was higher in areas with hot and dry climate, lower altitude, and extensive production of sugarcane.
Wesseling et al. ^11^ (2016) Longitudinal panel study with repeated measurements	Evaluation of renal function markers in 29 sugarcane cutters Nicaragua	A 9% decrease in the glomerular filtration rate, increased creatinine and serum urea and NGAL after 2 months of work.
Wesseling et al. ^63^ (2016) Cross-sectional study	Evaluation of risk factors for CKD among sugarcane cutters, construction workers, and small farmers Nicaragua	Thermal stress, dehydration, and kidney failure were the most common findings among sugarcane cutters and increased uric acid was associated with reduced renal function.
Wijkström et al. ^46^ (2017) Case series	Renal histopathological evaluation of 19 sugarcane workers Nicaragua	16 renal biopsies presented glomerulosclerosis, glomerular hypertrophy, chronic glomerular ischemia, tubulointerstitial damage, and mild vascular alterations.
Adesiyun et al. ^64^ (2010) Cross-sectional study	Prevalence of toxoplasmosis, leptospirosis, and brucellosis in sugarcane workers on the island of Trinidad	High risk of acute toxoplasmosis and, to a lesser extent, leptospirosis.
Carvalho-Junior et al. ^65^ (2012) Longitudinal study	Health-related quality of life assessment of 44 sugarcane cutters from the West of the State of São Paulo, Brazil	Quality of life, measured by questionnaire, was reduced at the end of the harvest period.
Priuli et al. ^66^ (2014) Longitudinal study	Stress levels and prevalence of physical and mental symptoms in 114 sugarcane workers State de São Paulo, Brazil	The work process of the cane cutter can cause stress, symptoms of burn out, exhaustion, physical and psychological symptoms after the harvest period.
Ferreira-Ceccato et al. ^67^ (2014) Cross-sectional retrospective descriptive study	Frequency of sick leave due to occupational diseases by sugarcane cutters State de São Paulo, Brazil	Musculoskeletal diseases followed by respiratory diseases at the end of the harvest were more prevalent.
Abrahão et al. ^16^ (2012) Cross-sectional study	To evaluate the impact of glove use on the safety, efficacy, and comfort of 82 sugarcane cutters State de São Paulo, Brazil	Results reveal general inadequacy of gloves used due to lack of adhesion to the machete, inadequacy in size, and hardening of the glove by contact with sucrose and ashes present in sugarcane.
Dalfré et al. ^68^ (2007) Cross-sectional study	Occurrence of fungi in the ocular conjunctiva of 100 sugarcane cutters Minas Gerais, Brazil	Of the 100 workers evaluated, 64 presented one or more genera of fungi, with higher incidence in the more advanced age groups.
Amre et al. ^69^ (1999) Case-control study	Investigate the risk of lung cancer among sugarcane producers Maharashtra, India	Risk of lung cancer doubled in workers on sugarcane farms.
Coble et al. ^70^ (2003) Case-control study	The relationship between occupational exposures and oral cavity or pharynx cancer (n = 367) Puerto Rico	High cancer risks were observed among sugarcane producers and in individuals with high cumulative exposure to solvents.
Bosso et al. ^47^ (2006) Longitudinal study	To evaluate the concentrations of 1-hydroxypyrene in the urine of 39 sugarcane workers State de São Paulo, Brazil	The level of 1-OHP in urine was 9 times higher in exposed workers compared to those not exposed.
Martinez-Vanezuela et al. ^48^ (2015) Cross-sectional study	To determine the chromosomal damage in oral mucosa in workers exposed to burnt sugarcane in Mexico (n = 60) compared to non-exposed workers (n = 60)	Higher presence of chromosomal micronuclei in oral mucosa and nuclear abnormalities in exposed workers, compared to non-exposed individuals.
Silveira et al. ^71^ (2013) Cross-sectional study	Genotoxic effect on burned sugarcane cutters (n = 23), compared to the control population (n = 30) Barretos, SP, Brazil	The frequencies of micronuclei in the cells of the mouth and blood were higher in sugarcane cutters.
Alessi et al. ^55^ (1997) Qualitative study	Assessment of the work process in sugarcane cutter’s health in Ribeirão Preto, SP, Brazil	Daily exposure of cane cutters to physical, chemical, and biological loads, translates into several diseases, traumas, or accidents related to them.
Alves ^17^ (2006) Review study	To analyze the processes of production and working day in the manual cutting of sugarcane State de São Paulo, Brazil	Cane cutters’ deaths related to overwork and payment per production.
Anchieta Messias et al. ^56^ (2012) Cross-sectional exploratory study	Evaluation of the posture in the work of a group of sugarcane cutters Pontal do Paranapanema, SP, Brazil	The movements and postures adopted during the work can predispose the cutters to repetitive strain injuries.
Galiano et al. ^72^ (2012) Qualitative study	Evaluation of the reasons of young people seeking work as sugarcane cutters and how they perceived their working conditions and health repercussions (n = 14) Ribeirão Preto, SP, Brazil	The data suggest that the migration of young workers in search of work was not an option, but the only alternative to the reality in their region of origin. They have expressed hopelessness about their prospects and concern about the possible consequences for their health.
Moraes et al. ^73^ (2013) Cross-sectional study	To characterize the socioeconomic profile, the motivation, the perception of the impacts of the work on health, and the relationship with the health system of migrants who cut sugarcane Mendonça, SP, Brazil	The majority of the workers (90%) came from the Northeast region of Brazil; were between 18 and 30 years old; 86% considered the salary and formal employment the motivators for this type of work; 92% considered their health good, although 48% felt some type of body pain attributed to work fatigue, and 87% self-medicates.
Phajan et al. ^37^ (2014) Cross-sectional analytical study	Prevalence and factors associated with work-related musculoskeletal disorders among 540 sugarcane workers Thailand	The prevalence of musculoskeletal disorders in the 7 days before the interview was 83%. The associated factors were: repetitive movements, inadequate postures, and vigorous efforts.
Ribeiro ^8^ (2010) Qualitative descriptive study	To portray the situation in which rural workers live in Macatuba, SP, observing their economic and social conditions, in the context of the implementation of the law that prohibits the burning of sugarcane and the mechanization of cutting, which bring an end to this type of employment (n = 40)	We evaluated 27 men and 13 women who received wages according to productivity. Part of them agreed with the ban to burnings because it eliminates much of the pollution they breathe, improving their quality of life.
Rocha et al. ^57^ (2007) Qualitative descriptive study	Assessment of individual, social, labor, and environmental factors predisposing to illness in 39 sugarcane cutters State de São Paulo, Brazil	The main individual determinants of illness were physical exertion and accelerated work rate, intense heat, dust, soot, and presence of venomous animals. Poverty is the main social determinant of illness.
Rocha et al. ^9^ (2010) Exploratory study with a quantitative approach	To analyze the work and life situations that may offer health risks to 39 workers involved in the manual and mechanized cutting of sugarcane State de São Paulo/Brazil	During work, workers are exposed to long daily shifts and work environments with multiple health hazards, and respiratory, musculoskeletal, psychological, and accidental impacts.
Rosa e Navarro. ^74^ (2014) Qualitative descriptive study	A total of 13 sugarcane cutter migrants were evaluated to understand the profile of the workers and to investigate their working conditions Ribeirão Preto, SP, Brazil	The workers are hired according to their capacity of production, physical resistance, and disposition of subordination to superiors. The production gain implies overexploitation of the labor force, which has repercussions on health conditions.
Scopinho et al. ^75^ (1999) Qualitative descriptive study	Evaluation by interviews conducted in the field, the consequences of mechanized cutting of sugarcane State de São Paulo, Brazil	The use of mechanical harvesters reduces physical, chemical, and mechanical labor loads, but accentuates mental and physiological loads. Observed reduction in the number of accidents at work, but increased severity.
Vilela et al. ^13^ (2015) Longitudinal study	Study of determinants that intensify the workload and affect the health of 40 sugarcane cutters Piracicaba, SP, Brazil	The accelerated pace of work associated with payment per production is the main factor responsible for the increase in the physical exhaustion among workers.
Bitencourt et al. ^49^ (2012) Case study	Contribution of the climate to the occurrence of 14 deaths of sugarcane cutters State of São Paulo, Brazil	The precarious social, economic, and working conditions of these workers do not allow us to point out the atmospheric factor as the predominant cause of the deaths.
Bodin et al. ^50^ (2016) Longitudinal study with repeated measures	To assess the feasibility of providing an intervention (adequate water replacement and programmed rest periods) during sugarcane cutting to avoid heat stress and dehydration without decreasing productivity (n = 60) El Salvador	Post-intervention water consumption increased by 25%. The symptoms associated with thermal stress and dehydration decreased. Daily individual production increased from 5.1 to 7.3 tons/person/day.
Crowe et al. ^38^ (2010) Observational and exploratory study	Conditions of heat stress in 130 sugarcane workers in Costa Rica in the pre-harvest period.	Risk of thermal stress for workers in pre-harvest tasks, which occurs even during periods of less intensive work.
Crowe et al. ^15^ (2013) Cross-sectional study	To describe the working conditions and quantify the heat exposure by sugarcane cutters (n = 105) Costa Rica	Cane cutters perform strenuous work under high temperatures, with no recommended breaks.
Crowe et al. ^10^ (2015) Cross-sectional study	Prevalence of complaints of heat exposure and health effects by 106 sugarcane cutters compared to 63 controls, workers in other activities Costa Rica	Symptoms of heat and dehydration (headache, tachycardia, cramps, fever, nausea, dizziness), hand or foot edema, and dysuria were more frequent in cane cutters than controls.
Roscani et al. ^51^ (2017) Cross-sectional study	Estimates of thermal overload in sugarcane cutters over a period of four years State de São Paulo, Brazil	The estimated IBUTG values exceed the limits of tolerance in the areas of sugarcane activity.
Miranda et al. ^78^ (2012) Cross-sectional study	Prevalence of actinic cheilitis in 1,950 sugarcane cutters exposed to the sun, compared to the control group (n = 150)	The prevalence of 9.2% (n = 141) of actinic cheilitis was observed among the population that had been exposed to the sun. However, no cases were found among the individuals in the control group.
Cortez et al. ^52^ (2009) Longitudinal study with repeated measures	Evaluation of the effect of hydration on the increase of labor productivity in workers exposed to high temperatures (n = 22) Nicaragua	Workers with higher water consumption increased production from 5.5 to 8 tons/day of cut cane.
Chiarello et al. ^53^ (2006) Longitudinal study with repeated measures	Impact of the use of protein and electrolyte supplements on weight and body composition of 15 sugarcane cutters Serrana, SP, Brazil	Observed reductions in body fat percentage and improvement in hydration at the end of the sugarcane harvest and 8 months after the beginning of the diet.
Florêncio et al. ^76^ (2008) Cross-sectional study	Evaluation of dietary pattern, nutritional status, and stature of 62 sugarcane cutters and possible associations with worker productivity Alagoas, Brazil	Workers with normal BMI were the most productive compared to those with low or overweight BMI. Taller individuals had higher productivity and higher energy intake.
Luz et al. ^77^ (2012) Longitudinal study with repeated measures	Evaluation of the evolution of the body composition of 30 cane cutters between the beginning and the end of the harvest Piracicaba, SP, Brazil	Significant loss of body fat and weight in the first half of the crop and elevation of creatine kinase over the harvest period.
Luz et al. ^54^ (2014) Semi-quantitative observational study	Description of working conditions, feeding, and hydration of 30 sugar cane cutters, under observation for 15 days Piracicaba, SP, Brazil	Workers drink 5 to 10 liters of water per day. Feeding during the harvest did not guarantee food and nutritional security. Work on manual cane harvesting is strenuous and payment per production can be an aggravating health factor.

IBUTG: Wet-bulb temperature-Globe Thermometer

### Work Environment and Organization

The process of manual cane cutting is an activity that imposes a high physical load on the cutter, since it requires the performance of vigorous, fast, and repetitive movements with a machete. In addition, there is the loading of the sugarcane bundles ^9^ . The manual cutting of cane requires the cutting of several canes near the ground and their gathering in bundles that weigh about 10 kg to 15 kg. The bundles are loaded for about two to five meters and arranged in rows to be picked up by the trucks that transport them to the mill for grinding ^34^ . Payment per production is an additional risk factor, as it induces a longer rate of work to guarantee a slightly better wage and a greater possibility of hiring in subsequent harvests ^8^ .

This study recorded the activities in films and allowed a more precise analysis of the activity in the cutting of burned sugarcane. During the workday, a worker that cuts 13 tons/day performs, on average, 3,100 spinal pushups, 3,500 machete blows, and 1,000 rotations of the lumbar spine ^13^ .

In addition, these workers are directly exposed to the pollutants generated by the burning of sugarcane and are constantly subject to adverse climatic conditions because the work is performed outdoors ^10^ ^,^ ^14^ ^,^ ^15^ . Work that requires physical exertion and high-temperature environment imposes risks of overload and thermal stress. This is aggravated by the use of overlapping clothing to reduce sun exposure, which hinders heat dispersion. In general, they do not take breaks, in disagreement with the Regulatory Standard – 15 of the Ministry of Labor and Employment – Order 3214/78, for thermal overload. For physical activity, the Standard provides a 15-minute working regime for 45 minutes of rest for Wet-Bulb Temperature – Globe Thermometer (IBUTG) values between 28°C and 30°C for activities with intense physical effort, as occurs with cutters, and work with IBUTG above 30°C ^13^ is not allowed.

The improper provision of personal equipment such as gloves and goggles, inadequate feeding and hydration and poor health conditions complement the environment and the work process to which these workers are subjected ^16^ ^,^ ^17^ .

### Respiratory Symptoms and Pulmonary Function

The inhalation of particulate matter released during the cutting of burned cane can affect the upper and lower airways, causing symptoms and respiratory diseases, as well as lung function impairment in the workers ^18^ .

Goto et al. ^19^ carried out a study involving 30 sugarcane cutters, with the main objective of evaluating nasal mucociliary transport, comparing the period of the harvest with the pre-harvest period. The authors did not observe differences in lung function between the periods. However, Prado et al. ^18^ developed a study with a larger number of workers and a control group, which evaluated respiratory symptoms and lung function. They found a higher prevalence of respiratory symptoms and a decrease in pulmonary function among sugarcane cutters during the harvest period compared to pre-harvest, with a decline in forced expiratory volume in the first second (FEV_1_), forced expiratory volume in the first second/forced vital capacity (FEV_1_/FVC), and forced expiratory flow (FEF25-75%), characterizing an evolution with a pattern of obstructive ventilatory disorder. A study by Ferreira-Ceccato et al. ^20^ evaluated the acute effects, i.e., four hours after the start of work, on the first day of harvest of burned cane and did not observe any complaint of nasal symptoms in any of the evaluated workers. However, the presence of symptoms is not a marker sensitive to the acute effect assessed by the study, thus limiting its interpretation.

Despite the increase in respiratory symptoms due to the burning of sugarcane, few studies have evaluated pulmonary function in cane cutters. Studies with more numbers of evidence are recommended to confirm these findings.

### Nasal Mucociliary Defense

The inhalation of pollutants increases with physical exertion, since it requires greater pulmonary ventilation. This implies an increased risk of nasal inflammation with increased production of proinflammatory cytokines ^8^ ^,^ ^21^ ^,^ ^22^ , with changes in nasal mucociliary clearance ^19^ ^,^ ^20^ .

Two studies evaluated the mucociliary clearance in cane cutters. The study by Goto et al. ^19^ , which evaluated 27 workers, showed that, in the harvest period, there was a reduction of 80% in the mucociliary clearance, with an increase in the saccharin transit time (STT) by 7.8 minutes and a reduction of 31% in the transportability of mucus. Ferreira-Ceccato et al. ^20^ evaluated the acute effects of exposure to particulate matter from the burning of sugarcane biomass in the nasal mucociliary clearance of sugarcane cutters. The evaluations occurred seven days before work on the sugarcane harvest and four hours later, on the first day of the sugarcane harvest. A significant reduction in STT was observed in the harvest period. The difference observed between the studies mentioned ^19^ ^,^ ^20^ can be explained by a mechanism similar to the one that occurs in smokers. In them, an increase in clearance at the beginning of tobacco consumption is observed, with shortening of STT as a defense response against aggression. Subsequently, with chronic exposure to tobacco smoke, changes in the rheology of mucus and hair cells, STT tends to increase^23–25^.

### Cardiovascular Changes

A study by Barbosa et al. ^14^ that evaluated 28 workers involved in sugarcane cutting during the harvest and pre-harvest observed a significant increase in blood pressure values during the harvest period. Monitoring of 24-hour systemic blood pressure showed an increase of 3.7 mmHg in systolic blood pressure during the harvest. The study also showed the effect of increased sympathetic activity, directly recorded in the fibular nerve, associated with elevated blood pressure. This suggests an autonomic nervous system imbalance effect as one of the mechanisms possibly implicated in elevated blood pressure. In addition to changes in blood pressure, the study by Barbosa et al. ^14^ also observed a significant decrease in the time of thrombin and prothrombin during the harvest. This indicates increased blood coagulation, which increases the risk of thromboembolic phenomena, changes that may be associated with both the inhalation of pollutants from the burning of the cane and dehydration.

Vilela et al. ^13^ , in a study with 40 cane cutters, evaluated cardiovascular load (CVL). This index is used to evaluate the physiological impact of the work because it corresponds to the percentage of the heart rate at work in relation to the maximum allowed heart rate. They observed a significant effect between the increase of productivity and CVL. Each increase in the cut of one ton of sugarcane was associated with an increase of approximately 0.81% in CVL. This finding evidences the impact of increased production on heart overload, i.e., work paid by productivity imposing greater cardiovascular risk.

The studies suggest an impact on the cardiovascular system of sugarcane cutters evidenced by increased blood pressure, increased cardiovascular load, changes in the autonomic nervous system, and changes in blood coagulability during the harvest compared to the pre-harvest.

### Inflammatory and Oxidative Stress Markers

The excessive physical exertion and exposure to heat and air pollutants to which workers are subjected during work in the cane harvest period may induce the development of oxidative stress and pulmonary and systemic inflammation^14,26–28^.

Prado et al. ^18^ observed a reduction of antioxidant enzymes: catalase, glutathione S-transferase (GST), glutathione reductase (GR), and glutathione peroxidase (GPx) in sugarcane cutters at the end of the harvest period compared to the pre-harvest period. Levels of malondialdehyde (MDA), a lipid peroxidation product of the cell wall, increased. When combined with the decrease in antioxidant enzymatic activity, this finding supports a chronic state of oxidative stress among cutters. However, Barbosa et al. ^14^ reported an increase in GST, GPx antioxidant enzymes in the burned sugarcane harvest period, probably in response to aggression, although it evidenced an increase in MDA levels. The authors suggested that both processes, an attempt to defend against oxidizing agents with increased protective enzymes and cell wall damage by membrane oxidation, may be concomitant. Differences in individual characteristics, genetic polymorphism, working conditions, eating, and living conditions may explain the different findings in the antioxidant markers. The cutters in the study by Barbosa et al. ^14^ lived in the São Paulo region of Sorocaba-Piracicaba, SP, and those involved in the study by Prado et al. ^18^ were all migrants from Paraíba and Pernambuco, who worked in the region of São José do Rio Preto, SP, only during the harvests.

Other manifestations caused by strenuous work ^14^ ^,^ ^18^ are associated with the elevation of muscle injury biomarkers such as creatine kinase (CK) and lactate dehydrogenase (LDH), and electrolyte changes compatible with work under conditions of physical overload and hydroelectrolytic imbalance ^29^ . The CK and LDH are biomarkers that may increase during situations of intense exercise, in which the cell membranes become more permeable and release various compounds into the blood, including myoglobin ^30^ . In the study by Santos et al. ^31^ , there was an acute increase in CK serum levels, which increased from 120 IU/l before the beginning of the workday to 360 IU/l at the end of a day’s work day. In the study by Barbosa et al. ^14^ , although of small magnitude, elevations in serum levels of CK and DHL were observed during the harvest in relation to the pre-harvest period, suggesting chronic muscle injury.

Elevated levels of CK, DHL, and myoglobin in plasma result from muscle injury due to intense and strenuous physical exertion, which can be aggravated in unfavorable environmental conditions ^32^ . In addition, muscle damage with rhabdomyolysis is a factor associated with the development of acute kidney injury (AKI)^33–36^, particularly in dehydration situations ^37^ ^,^ ^38^ . In addition to serum levels of CK and LDH, the excessive activity may induce increased ventilatory work with increased inhalation of nephrotoxic substances such as silica and metals ^39^ , increasing oxidative stress ^27^ and systemic inflammation ^40^ . Santos et al. ^31^ observed a significant increase in leukocyte counts as well as neutrophils at the end of a working day for cane cutters during the harvest period. This indicates an inflammatory response probably associated with strenuous work, under high temperatures and exposure to pollutants.

The studies assessed revealed the occurrence of oxidative stress, increased concentrations of biomarkers for muscle injury and inflammatory cells in the blood. This is possibly associated with excessive physical stress at high temperatures and exposure to air pollution by sugarcane cutters during the harvest period.

### Renal Effects

Health registries and research in Central America show the occurrence of a chronic kidney injury epidemic in rural workers^41–44^. It was first described by Trabanino et al. ^42^ and was renamed Mesoamerican nephropathy.

Despite several studies, its etiology has not been clarified. One of the hypotheses is that it can be caused by repeated episodes of acute renal damage due to daily dehydration associated with rhabdomyolysis, systemic inflammation, oxidative stress, genetic variations, and exposures to non-characterized pesticides ^42^ . Wesseling et al. ^11^ evaluated 29 sugarcane cutters in Nicaragua and found a significant decrease in renal function during the nine-week sugarcane cutting work. The estimated mean glomerular filtration rate decreased (9%, 10 mL/min), there was a significant increase in serum creatinine (20%), serum urea (41%), and four-fold elevation of lipocalin associated with neutrophils (NGAL), a biomarker for the early detection of renal damage. In the Santos et al. ^31^ study, which involved 28 sugarcane cutters evaluated before and after a working day at the end of the harvest period, there was a significant increase in urinary density, lower levels of serum sodium and fractional excretion of sodium (FeNa), as well as a significant increase in the hematocrit at the end of the working day, suggesting that the sugarcane cutters were dehydrated.

Dehydration, thermal stress, and volume depletion are known risk factors for the development of renal disease. In the study by Garcia-Trabanino et al. ^45^ , the high prevalence of a reduction in the glomerular filtration rate was consistent with the dehydration caused by strenuous work in hot and humid environments. The cause may be related to decreased renal blood flow, increased demand for tubular reabsorption, and increased levels of uric acid ^45^ .

Renal biopsies were performed in sugarcane workers in Nicaragua, and glomerulosclerosis, glomerular hypertrophy, signs of chronic glomerular ischemia, and tubulointerstitial damage and mild vascular alterations were found ^46^ . This gave biological plausibility to the hypotheses raised ^45^ .

Twelve studies reported impairment of renal function in sugarcane cutters. The following were found: decreased glomerular filtration rate, increased creatinine, urea, increased urinary density, and biomarkers for the early detection of renal damage. Work conditions cannot be ruled out as one of the factors that may have contributed to the chronic kidney disease epidemic in several Central American countries and some Asian countries, especially among sugarcane workers. However, the etiology and pathophysiology of the chronic renal injury epidemic in these workers is uncertain. We also do not know whether the repetition of acute injuries may be one of the causes associated with genetic characteristics, varied environmental exposures, and the use of anti-inflammatory drugs in the induction of chronic kidney disease.

### Exposure to Potentially Carcinogenic Agents and Genotoxic Effects

Three studies ^18^ ^,^ ^47^ ^,^ ^48^ carried out in sugarcane regions found increased values in the markers of exposure to aromatic hydrocarbons in workers involved in sugarcane cutting. A study involving 90 cutters showed that the concentration of 1-OH-Pyrene in the urine was 11 times higher in the harvest period than in the pre-harvest period ^18^ . This result was similar to those of Bosso et al. ^47^ , who evaluated 39 cane cutters and found a 10-fold higher concentration of 1-OH-Pyrene in the urine during the harvest compared to the pre-harvest period. Martinez-Vanezuela et al. ^48^ evaluated chromosomal damage in rural workers with sugarcane burned in Sinaloa, Mexico. We analyzed 1,000 buccal epithelial cells from 60 exposed and 60 unexposed workers (controls) to determine micronucleus frequencies and other nuclear abnormalities. The results indicated higher values of micronuclei and nuclear abnormalities in the exposed subjects compared to those not exposed. The burning of sugarcane, which generates polycyclic hydrocarbons, represents a genotoxic risk for sugarcane workers.

### Miscellaneous Effects

Studies have evaluated heat stress and dehydration in sugarcane cutters ^10^ ^,^ ^15^ ^,^ ^38^ ^,^ ^49^ ^,^ ^50^ . In the study by Crowe et al. ^10^ , symptoms associated with exposure to heat or dehydration (headache, tachycardia, cramps, fever, nausea, dizziness, hand or foot edema, and dysuria) have been reported at least once a week among the 106 workers evaluated.

Roscani et al. ^51^ , in a study that evaluated the risk of thermal overload in sugarcane cutters for four years in the State of São Paulo, observed that the IBUTG values exceeded the tolerance limits in about 7% of the days for heavy activity and at about 3% for moderate activity. These estimates contradict findings by Barbosa et al. ^14^ and Vilela et al. ^13^ However, this study ^51^ presents a limitation, since the IBUTG data were not recorded directly in the work field. The measurements were performed using IBUTG estimates using data provided by the INMET – National Institute of Meteorology’s network of meteorological surface stations, which may have underestimated the measurements.

Bodin et al. ^50^ evaluated the feasibility of providing intervention (adequate water replacement and scheduled rest periods) during sugarcane cutting to avoid heat stress and dehydration, without decreasing productivity. Post-intervention water consumption increased by 25%, the symptoms associated with thermal stress and dehydration declined, and daily cane production increased from 5.1 to 7.3 tons/person/day. Cortez et al. ^52^ also observed that workers with higher water consumption increased daily production from 5.5 to 8 tons of cut cane. In the study by Chiarello et al. ^53^ , after the use of protein and electrolyte supplements, the reductions in body mass index and percentage of body fat with lean mass maintenance were significant. In addition, there was an improvement in the hydration state of 15 sugarcane cutters during the harvest. These studies ^50^ ^,^ ^52^ ^,^ ^53^ , aimed at meeting employer rationality, increasing production and immediate wage gains, did not consider the acute and chronic harmful effects that are caused by the effort made to increase production. On the other hand, Luz et al. ^54^ , in a study which carried out direct observation of a fieldwork with 40 sugarcane cutters, concluded that the manual cutting of cane is strenuous and the payment per production can be an aggravating factor for health. The authors suggest that correct nutrition and hydration could minimize wear and tear and pain during work.

A review study by Alessi et al. ^55^ addressed the effect of the working process on sugarcane cutter health and suggests that the daily exposure of cane cutters to physical, chemical, and biological loads becomes a series of diseases, trauma or related accidents ^55^ . The movements and postures adopted during the work may predispose the cutters to injuries due to repetitive stresses or musculoskeletal diseases ^56^ . Rocha et al. ^57^ evaluated individual, social, and environmental factors predisposing 39 workers to illness and observed that the main determinants for illness were physical exertion, fast pace of work, living conditions, and poverty.

Most studies that address the environment and work organization are qualitative. This type of study is greatly relevant to qualify risks and problems raised by workers. However, there are limitations to better assess the risks and health effects on workers, as they are usually only conducted through interviews.

Despite the implementation of laws and measures that provide for the elimination of sugarcane burning, this practice is still carried out in several regions of Brazil and in several countries. Because this work is aggressive to health, even when the cut is made in non-burned cane, this activity must undergo changes to protect workers. These changes should include a review of production gain, elimination of pre-harvest burning, mechanization, and the existence of breaks at work. This process must be carried out with the participation of the workers so that the use of new technologies and the establishment of public policies that compensate for lost jobs may be properly implemented. In addition, there must be a qualification of workers, so that they may occupy the new jobs generated by mechanization.

## CONCLUSION

Work in the manual cutting of sugarcane, especially when the cane is burned, exposes workers to several risks responsible for health problems – respiratory, renal, cardiovascular, osteomuscular, ocular, and dermatological.
